# Quality-of-life assessment in autistic adults with lower support needs: gaps and emerging challenges

**DOI:** 10.3389/fpsyt.2026.1756393

**Published:** 2026-04-01

**Authors:** María Fernández, Dominika Zofia Wojcik, Emiliano Díez

**Affiliations:** 1 Departamento de Personalidad, Evaluación y Tratamiento Psicológicos, Universidad de Salamanca, Salamanca, Spain; 2 Instituto Universitario de Integración en la Comunidad (INICO), Universidad de Salamanca, Salamanca, Spain; 3 Instituto de Investigación Biomédica de Salamanca (IBSAL), Salamanca, Spain; 4 Departamento de Psicología Básica, Psicobiología y Metodología de las Ciencias del Comportamiento, Universidad de Salamanca, Salamanca, Spain

**Keywords:** adults, assessment tools, autism spectrum disorder, diversity, lower support needs, quality of life

## Abstract

Quality of life (QoL) has become a key construct in the planning of support for people with disabilities. In the case of autism spectrum disorder, particularly among adults with lower support needs, there is a notable lack of valid, sensitive, and culturally adapted tools to assess their well-being. Although this group often presents a functional cognitive and linguistic profile, they experience significant—and frequently overlooked—challenges in areas such as emotional regulation, social interaction, access to services, and community participation, all of which substantially impact their QoL. This mini-review examines the current state of knowledge in this field, addressing (1) conceptual models of QoL relevant to disability; (2) the main clinical, personal, and contextual determinants of QoL in adults with autism; (3) the limitations of currently available QoL instruments; and (4) the urgent need for new methodological developments participatory and sensitive to the specificities of autistic experience. Conceptual, psychometric, and representativeness gaps are identified, particularly regarding the additional challenges faced by women, people with diverse gender identities, and other groups historically underrepresented in autism research. In this regard, the present work highlights the need to advance toward more inclusive assessment models, sensitive to the profiles of individuals with autism without intellectual disability—grounded in participatory approaches that acknowledge the intersection of autism, gender, and other dimensions of diversity. The aim is to foster a more accurate understanding of well-being in this population and to advance person-centered practices and interventions that are more appropriately tailored to their specific and unique needs.

## Introduction

Autism spectrum disorder (ASD)^1^ is a neurodevelopmental condition characterized by difficulties in social communication and the presence of restricted and repetitive patterns of behavior (1, 2). Although it has often been conceptualized as a unidimensional construct, the term “spectrum” highlights the marked variability in clinical manifestations and support needs, which may range from high to lower levels of support, with diverse life trajectories and outcomes depending on individual profiles, life stage, context, and lived experiences. Recent estimates place the global prevalence of ASD between 0.79% and 1% (3, 4), with a high clinical, social, and functional impact, making it a priority for research and intervention.

Within the spectrum, autistic adults with lower support needs, known as Level 1 ASD (1), have been underrepresented in the literature, especially regarding their quality of life (QoL), a construct that reflects the desired living conditions of individuals in relation to fundamental needs, serving as a social framework that guides person-centered practice and evaluation in disability contexts (5). Indeed, although these autistic adults generally present functional cognitive and communicative profiles, many continue to face significant challenges in adulthood. Even in the absence of intellectual disability (ID), autistic adults may present difficulties in cognitive empathy and social interaction (6), which may be associated with poorer social functioning and, in some cases, with lower QoL. This suggests that cognitive or linguistic competence alone does not guarantee an obstacle-free life experience (7–9).

The spectrum also encompasses broad sociodemographic and cultural diversity, including subgroups historically underrepresented in research, such as women (8, 10, 11), individuals with diverse gender identities (12), people of migrant or culturally diverse backgrounds (13, 14), and adults across different life stages and socioeconomic contexts (15). These intersectional modulators shape diagnostic visibility, stigma experiences, levels of camouflaging, opportunities for participation, etc., and together impact QoL. Intersectional domains influence autistic individuals’ subjective experiences and affect structural barriers in their lives. In this regard, contextual factors play a decisive role in life trajectories and QoL of adults with autism (16, 17).

Late diagnosis is one such contextual factor, as it may restrict access to specialized resources and hinder the reinterpretation of past experiences (18), with significant implications for identity construction and perceived QoL. It has also been associated with higher levels of social anxiety, loneliness, and lower perceived social support (19). It is estimated that between 59% and 72% of autistic adults in the United Kingdom remain undiagnosed (20), revealing a substantial gap in detection and follow-up and leaving autistic individuals without adequate support during critical life transitions, such as attaining functional independence, making decisions, or accessing employment (15, 21).

These diagnostic and service gaps intersect with structural barriers that hinder access to essential resources for an autonomous and fulfilling life, such as mental healthcare, independent living, and—most prominent of all—employment. Even without ID, employment rates remain very low and are linked to difficulties achieving autonomy (22–27). Employment instability and lack of support often coexist with mental health problems, particularly anxiety and depression, which increase dependence on assistance and shape a psychosocial vulnerability profile that limits self-determination—one of the key domains in the QoL model (7, 22, 26). These limitations directly affect social participation, another QoL dimension, and translate into lower QoL compared with the general population, especially when formal and informal supports are insufficient (17, 28–30). Recent reviews identify employment and mental health as key predictors of QoL, underscoring the need for specific and sustained interventions in these areas (21, 31, 32). Likewise, high emotional vulnerability and difficulties exercising self-determination have been related to the lack of adequate social and mental health supports (33, 34).

Taken together, these findings highlight the need to move toward a comprehensive and multidimensional characterization of autistic adulthood, particularly among individuals with lower support needs. This approach requires considering contextual, sociodemographic, and cultural determinants influencing QoL and promoting a dynamic understanding of functioning that transcends traditional clinical indicators. From this perspective, QoL constitutes a particularly useful theoretical framework for articulating this comprehensive understanding. However, as shown in previous studies, contemporary QoL frameworks and related instruments have notable validity and sensitivity limitations when applied to autistic adults with lower support needs. These tools often fail to fully capture aspects of everyday experience and display differential item functioning (DIF) across subgroups (e.g., by gender), which contributes to the invisibility of part of their lived realities (16, 35–38). For example, commonly used QoL instruments may interpret employment, social participation, or independence as unequivocal indicators of well-being, while overlooking the emotional and cognitive costs associated with sustained social camouflaging or chronic sensory overload. Likewise, domains such as emotional well-being often fail to distinguish between apparent adaptation and internal distress, particularly among autistic women and gender-diverse individuals. As a result, these tools may overestimate QoL while underdetecting clinically relevant vulnerability. This misalignment between measured domains and lived experience compromises content validity and reduces the sensitivity of current instruments to detect meaningful changes in well-being among autistic adults with lower support needs.

The present work synthesizes recent evidence on QoL in autistic adults with lower support needs, identifying conceptual and methodological gaps and outlining future research directions. To this end, the main conceptual models of QoL in the field of disability are reviewed, along with the determining factors that influence it and the current state of assessment instruments available for the adult autistic population, to lay the groundwork for more inclusive, accurate, and culturally sensitive evaluation and intervention strategies.

This article is a narrative mini-review rather than a systematic review or meta-analysis and may involve selection bias or incomplete coverage. However, we focus on peer-reviewed empirical and conceptual work published mainly over the last decade that addresses QoL in autistic adults without ID or with lower support needs. We prioritize studies that (a) use QoL or closely related constructs as an outcome; (b) show self-report data from autistic adults, while also considering proxy reports and objective indicators; and (c) examine the role of gender, camouflaging, stigma, and other contextual or intersectional determinants. Our aim is not to provide an exhaustive mapping of the literature, but to highlight key conceptual, methodological, and representational gaps and to outline priorities for future assessment and intervention. The literature considered was identified through targeted searches in major databases commonly used in health and psychological research (e.g., PubMed, PsycINFO, and Scopus). The search was not restricted to psychiatry or mental health journals and included contributions from disability studies, rehabilitation, psychology, and related interdisciplinary fields. The review focused primarily on articles published in English and Spanish. Although the reviewed studies are international in scope, with a predominance of research conducted in Europe and North America, no geographical restrictions were applied *a priori*.

## Conceptual models of quality of life in disability

Historically, QoL frameworks have mainly approached health-oriented perspectives [e.g., (39)], focusing on physical indicators; life satisfaction approaches [e.g., (40)], which prioritize subjective perceptions of well-being; and family context models [e.g., (41)], but they do not fully fit conditions such as ID, brain injury, or autism. The Schalock and Verdugo (5) QoL model addressed these limitations as it defines QoL as a desired state of well-being that is multidimensional, encompassing both objective and subjective components, and influenced by personal and environmental variables. It integrates eight domains: emotional (feeling calm and secure), physical (enjoying good health and healthy habits), and material well-being (having adequate resources and living conditions); interpersonal relationships (maintaining positive and supportive social connections); personal development (learning, growing, and achieving self-fulfillment); self-determination (having the ability to make choices and decisions about one’s own life); social inclusion (actively participating in community life); and rights (being treated with equality, respect, and dignity). Its cross-context applicability, cultural sensitivity, and person-centered emphasis have favored its international adoption as a theoretical framework for the assessment of QoL in people with disabilities and its subsequent implementation in organizational and service contexts (42, 43).

It also acts as a bridge between the values and rights promoted by international frameworks (such as the Convention on the Rights of Persons with Disabilities and the Sustainable Development Goals) and evidence-based practice. This model led to development of several indicators, assessment tools, person-centered intervention guidelines (44), validated scales and questionnaires all used for individual planning, intervention evaluation, and the design of inclusive policies (e.g., Gencat Scale, INICO-FEAPS). However, most of these instruments have been designed primarily for individuals with ID or high support needs (45, 46), or for children and youth (47), leaving the specific needs of autistic adults without ID largely unaddressed. This creates issues of applicability and ecological validity, since key experiences—sensory overload, social camouflaging, and identity congruence—are not adequately captured (10, 48). Importantly, this limitation not only reflects gaps at the individual item level but also points to the fact that experiences are not explicitly embedded within the underlying conceptual models of QoL on which such instruments are based. For instance, existing instruments rarely differentiate between meaningful social connection and socially demanding interactions sustained through camouflaging, nor do they adequately assess the cumulative impact of sensory overload on daily functioning. Consequently, the resulting QoL profiles may fail to reflect the subjective priorities and stressors that shape everyday well-being in autistic adulthood, limiting the ecological validity of these assessments.

While the framework of Schalock and Verdugo (5) provides a robust foundation for evaluating well-being through an integral, person-centered approach, its adaptation to the autistic context requires incorporating nuances that reflect experiences inherent to this condition. Understanding QoL in autistic adults requires examining how clinical, personal, social, structural, and intersectional factors interact and shape diverse life trajectories, and the next section reviews those most relevant for adults with lower support needs.

## Factors influencing quality of life in adults with autism spectrum disorder

The determinants of QoL in autistic adults without ID can be grouped into five broad domains: clinical, personal or psychological, social or environmental, contextual or structural, and sociodemographic/intersectional. Co-occurring mental health conditions, particularly anxiety and depression, stand out as the most consistent predictors of lower QoL (21, 31). Recent evidence reinforces this conclusion, indicating that depression represents the primary factor associated with lower QoL, even surpassing anxiety in predictive power (49). In contrast, variables traditionally used to characterize autism, such as intelligence quotient or diagnostic severity, show no robust or consistent associations with QoL in adulthood (17, 30, 50).

Personal or psychological factors also play a central role in the QoL of autistic adults. Intrapersonal processes such as acceptance of the diagnosis, development of a positive autistic identity, autonomy in decision-making, and the use of adaptive coping strategies are associated with higher levels of QoL. Viner et al. (18) synthesize these elements into five interrelated domains: diagnostic understanding, social connection, autonomy, structural barriers, and stigma. At this level, social camouflaging is also relevant; it refers to the suppression of autistic behaviors and the deliberate adoption of neurotypical behaviors to facilitate social acceptance (10, 51). While useful in the short term, it has some long-term psychological costs, including emotional exhaustion, depression, chronic anxiety, loss of identity coherence, and increased suicide risk (10, 51, 52).

Social and environmental factors constitute an essential component of QoL. Loneliness, lack of community participation, and limited access to adequate supports, negatively impact QoL (53). Moreover, meaningful relationships, perceived social support, both formal and informal, and social connectedness predict higher QoL levels (18, 54). Likewise, active community engagement and employment are linked to higher well-being and psychosocial adjustment (33, 55, 56).

Among contextual or structural factors, age at diagnosis emerges as a key modulator of QoL. Early diagnosis tends to show better psychological adjustment and well-being, whereas late diagnosis results in cumulative psychological and social costs that impact life trajectories and access to specialized supports (18, 20). These differences are compounded by persistent structural barriers such as discontinuity of services in adulthood, imbalanced quality of healthcare systems, and limited access to educational and employment opportunities (57, 58).

Regarding sociodemographic factors, certain social positions, such as gender, operate as intersectional modulators that decisively shape the experience of QoL in autism, particularly among individuals without ID. Autistic women tend to receive diagnoses later in life, report higher levels of anxiety and depression, and exhibit poorer physical and mental health indicators compared with men (8, 19, 59). These differences are largely related to the frequency and impact of social camouflaging, as women report significantly higher levels of it than men, leading to delayed diagnosis and contributing to underdiagnosis and invisibility of their needs (e.g., (38, 60, 61)). From an intersectional perspective, gender does not operate in isolation but interacts with clinical, personal, and social factors, influencing the expression of autism, life trajectories, and access to support. These dynamics affect not only autistic women’s lived experiences but also how these experiences are captured in research and clinical practice, as some QoL assessment tools have been shown to contain gender biases that tend to underestimate self-reported well-being (38).

Finally, social stigma emerges as a cross-cutting factor and one of the most consistent negative predictors of QoL in autistic adults (18, 57, 62, 63). Its impact manifests at multiple levels: At the individual level, it is associated with greater depression, anxiety, and lower self-esteem (64); at the relational level, it limits opportunities for social participation and erodes perceived support, thereby increasing social isolation (65); and at the structural level, stigmatizing attitudes reinforce institutional barriers that restrict access to services, education, and employment (66, 67). Moreover, these dynamics interact with gender and other social positions, amplifying vulnerability and invisibility among specific groups within the spectrum (65).

Within existing QoL frameworks, some of these determinants are typically addressed indirectly through broad domains such as emotional well-being, social inclusion, or self-determination. Although many of the determinants identified are partially represented within existing QoL frameworks, others remain insufficiently operationalized in current assessment tools. Factors such as social camouflaging, internalized stigma, identity congruence, and structural barriers are rarely assessed directly, despite their documented impact on well-being. As a consequence, current instruments provide only a partial operationalization of empirically established determinants of QoL, limiting their capacity to capture the full range of factors shaping well-being in autistic adults.

## Current status of quality of life assessment instruments in autism spectrum disorders

Most studies continue to employ generic instruments developed for the general population, such as the WHOQOL-BREF (68), without conceptual or cultural adaptations for adults with autism. This lack of specificity leads to problems of content validity and limits the sensitivity of these tools to detect key aspects of autistic experience, such as social camouflaging, autistic identity, stigma, or social barriers (69). Consequently, the resulting well-being profiles may be unrepresentative or even distorted, as they fail to adequately reflect the priorities and challenges specific to this population.

In the Spanish context, used here as an illustrative case of broader cross-cultural and linguistic challenges in QoL assessment, widely used scales such as INICO-FEAPS (45) or San Martín (46) were designed for individuals with ID, whereas the KidsLife-TEA scale (47) targets children and adolescents. Internationally, the development of the Autism Spectrum Quality of Life (ASQoL) (35) represented an important milestone, as it included items derived directly from the lived experiences of autistic adults. However, subsequent studies have questioned its structural validity and revealed potential gender biases in some items (38), compromising its measurement invariance across groups.

Another methodological challenge lies in the discrepancy between self-report and proxy-report measures. While self-report is essential for capturing the subjective perspective of well-being, interpretation biases have been noted for some items. Conversely, third-party reports can complement assessment but risk introducing biases. Corbera et al. (6) proposed integrating both approaches, but this must be balanced with ensuring autistic people’s self-determination through accessible self-report formats (e.g., plain language, visual supports, and contextualized items) that reflect their voices.

In addition, future instrument development must consider the balance between comprehensiveness and practical usability. Because QoL is inherently multidimensional, instruments that attempt to capture all relevant domains may become lengthy and demanding for respondents. This may limit their feasibility in routine clinical or community settings, where shorter and more accessible tools are often necessary to ensure completion and practical utility.

Low participation of autistic people in instrument design and development has been identified as a critical issue. Only approximately 12% of studies include their perspective (36) despite the fact that it has been shown that active inclusion of autistic individuals as co-researchers enhances the relevance, clarity of instruments, and their conceptual and cultural validity (70, 71). Participatory approaches situate research within real-world contexts, increase the social relevance of findings, and strengthen trust and collaboration between researchers and the autistic community (72). Finally, limitations persist in the psychometric rigor of current tools. The lack of invariance analyses across relevant subgroups (e.g., gender, age, and support level) may lead to misinterpretation of results across different profiles within the spectrum (38, 73), whereas the scarcity of longitudinal studies constrains knowledge about sensitivity to change, limiting the use of these tools to monitor the effectiveness of interventions or life-course trajectories (21). Addressing these limitations is essential to developing valid, sensitive, and comparable instruments that can assess the real impact of person-centered interventions and link QoL outcomes to evidence-based support and policy (44).

## Discussion

The assessment of QoL in autistic adults remains an emerging field that still presents conceptual, methodological, and applied gaps. This mini-review highlights that, although solid conceptual models of QoL have guided assessment and support provision in the field of disability, their application to autistic adults with lower support needs is still partial. In practice, there is a mismatch between these models and the complexity of autistic experience, which is shaped by the interaction of clinical, personal, social, structural, and intersectional factors. As a result, there is a persistent lack of valid, specific, and culturally adapted tools that, even when grounded in robust theoretical frameworks (e.g., Schalock & Verdugo), can accurately capture the experiences, priorities, and barriers that are unique to this group. The main challenge is not only to develop new scales but to incorporate often overlooked domains (e.g., camouflaging, identity congruence, and sensory experiences) while integrating objective indicators and subjective perceptions and ensuring construct validity, gender invariance, and cultural sensitivity.

The evidence reviewed regarding the determinants of QoL in autistic adults converges on several key implications for research and professional practice. Enhancing QoL in this population requires a comprehensive approach that encompasses both individual and contextual factors. At the individual level, this includes identifying and addressing co-occurring mental health conditions—particularly anxiety and depression—promoting autonomy and positive autistic identity, and strengthening stable social support networks. At the contextual level, reducing stigma, facilitating early diagnosis, and ensuring continuity and adequacy of supports are indispensable conditions for inclusion and well-being. Some of these dimensions can be directly measured within QoL frameworks, whereas others operate as external determinants that shape QoL and therefore must be explicitly incorporated into the design, adaptation, and validation of future instruments. Systematic integration of these elements would allow for more accurate, inclusive, and context-sensitive measurements of QoL in autism.

In line with this, the development of new assessment tools should adopt participatory methodologies that involve autistic individuals from the earliest stages of the process, promoting their active role as co-researchers and ensuring validation across diverse subgroups (e.g., by gender, age, support level, and sociocultural background). Likewise, the integration of accessible technologies (e.g., adapted digital platforms, screen readers, or visual-support applications) can facilitate self-assessment and longitudinal monitoring, thereby increasing autonomy and self-regulation of well-being (71, 74). Ensuring robust psychometric properties—such as content and construct validity, internal reliability, measurement invariance, and sensitivity to change—is essential for these tools to provide a solid foundation for clinical, organizational, and policy decision-making (75). At the same time, the development of future QoL instruments must also address the practical challenges inherent to operationalizing a multidimensional construct such as QoL. Capturing the complexity of autistic experiences requires comprehensive conceptual coverage, yet instruments that are excessively long may generate respondent burden and reduce completion rates, particularly in clinical or community-based settings. Therefore, future scale development should seek to balance conceptual depth with practical feasibility, considering factors such as scale length, accessibility, and ease of administration to ensure that QoL measures remain both meaningful and usable across research and applied contexts.

**Figure 1:**
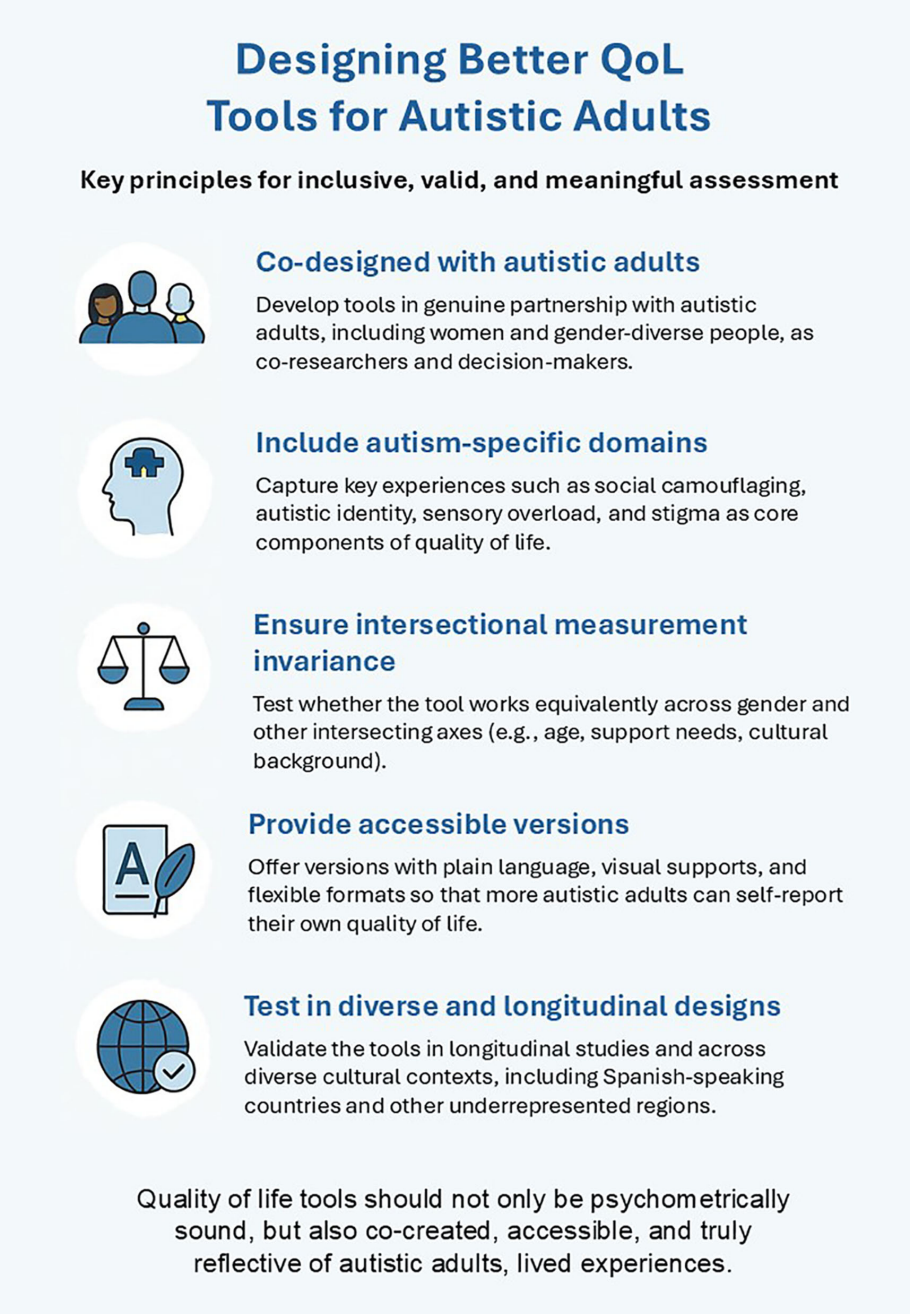
Key principles for designing inclusive quality-of-life assessment tools for autistic adults.

The absence of valid, autism-specific QoL instruments for adults with lower support needs has direct practical consequences in clinical settings: the accurate identification of needs and the planning and monitoring of individualized supports. In research, it restricts the possibility of conducting comparable longitudinal follow-ups and rigorously evaluating intervention outcomes. In organizational and policy contexts, it limits the capacity to design and evaluate evidence-based policies that respond equitably to the diversity of profiles within the spectrum. From this analysis, several priorities emerge: consolidating culturally adapted and validated scales in Spanish, as a part of broader efforts to improve linguistic and cultural accessibility in QoL assessment across contexts; addressing validity, invariance, and gender-bias issues present in current tools; and promoting longitudinal and multicenter studies to examine life-course QoL trajectories and the long-term impact of supports. Achieving these goals (for visual summary see **Figure 1**) will require participatory, collaborative, and translational dialogue between research, public policy, and community services, ensuring that QoL outcomes are translated into concrete improvements in support planning and into the reduction of structural inequalities. This mini-review should be interpreted in light of several limitations. As a narrative review, it may involve selection bias and does not provide exhaustive coverage of the literature. In addition, the review relies on published peer-reviewed studies and encompasses a heterogeneous body of work that varies in conceptualizations of QoL and assessment approaches. Moreover, the geographical representation of the available evidence is uneven, which may limit the generalizability of some conclusions. Nevertheless, the review offers an integrative synthesis of conceptual, methodological, and applied challenges in QoL assessment, with a specific focus on autistic adults with lower support needs. Its emphasis on participatory approaches and ecological validity represents a key strength with direct implications for research, clinical practice, and policy development.

Ultimately, advancing this field represents a crucial step not only toward optimizing QoL assessment but also toward ensuring the exercise of rights, social inclusion, and equity within support systems. A research agenda that combines psychometric rigor, diversity of perspectives, and the active participation of autistic people can help to highlight the heterogeneity of autistic adulthood, reduce stigma, and drive more just and inclusive evidence-based policies.

## Footnotes

1 This paper uses both person-first (e.g., “person with autism”) and identity-first (e.g., “autistic person”) language to respect diverse preferences within the autistic community. Medical terminology (ASD) is used only when referring to diagnostic criteria or clinical contexts.
